# Power of public health advice: Effectiveness and spillover effects of federal vaccine recommendations

**DOI:** 10.1016/j.puhip.2025.100667

**Published:** 2025-10-14

**Authors:** Junying Zhao, Ahmed El Fatmaoui, Mahla Shourian, Bethanie Lor, Pallab K. Ghosh

**Affiliations:** a Department of Health Administration and Policy, University of Oklahoma Health Sciences, 801 NE 13th Street CHB 359, Oklahoma City, OK, 73104, USA; b Department of Economics, University of Oklahoma, 308 Cate Center Dr, Norman, OK, 73019, USA

**Keywords:** Federal recommendation, H1N1, Influenza, Insurance, Policy, Spillover effect, Vaccine

## Abstract

**Objectives:**

This paper focuses on the national-level, inexpensive, demand-side vaccine policy—federal recommendations. It evaluates the effectiveness of 2008 and 2010 influenza vaccine recommendations, the spillover effect of the 2009 H1N1 vaccine recommendation on influenza vaccination, and heterogeneous policy effects across individual characteristics.

**Study design:**

Observational study with before-after comparison assessing changes in vaccination following policy implementation.

**Methods:**

We used nationally representative 2004–2015 NHIS data on 77,361, 23,653, and 238,866 individuals in age groups targeted by the 2008, 2009, and 2010 policies, respectively. Using the Linear Probability Model with fixed effects, we estimated policy effectiveness, spillover effects, and heterogeneous effects across individual characteristics.

**Results:**

Both 2008 and 2010 influenza vaccine recommendations boosted influenza vaccination likelihood by 20.9–26.5 % among children and 5.2–6.6 % among older adults. The 2009 H1N1 vaccine recommendation had a positive spillover effect, with a 5.7–9.8 % increase in influenza vaccination likelihood among younger adults. Low influenza vaccination likelihoods exist across demographic and socioeconomic characteristics: Children uninsured or privately insured, White, and low-income; Adults uninsured or publicly insured, White, African and Hispanic American, male, childless, self-reported poor or excellent health, low-educated, and low-income.

**Conclusions:**

Future policies may address the cost barriers faced by the uninsured, and multi-level non-cost barriers experienced by privately insured children and publicly insured adults. Future policies may consider extending beyond the federal recommendation, such as implementing simultaneous anti-poverty policies, to achieve minimum coverage and utilize the spillover effects of one vaccine policy to maximize coverage of other vaccines. Future research may investigate potential policy spillover effects among influenza, COVID-19, RSV, and new vaccines.

## 1 Introduction

Coverage of life-saving and cost-saving vaccines is influenced by multi-level and multi-type determinants that remain inadequately evaluated. Determinants vary from individual to interpersonal, organizational, community, and policy levels. Policies vary from local to national levels, expensive to inexpensive, demand-side to supply-side types [1]. This paper focuses on the national-level, inexpensive, demand-side policy, particularly federal vaccine recommendation.

Spillover effects of one vaccine policy (e.g., state-mandated Tdap vaccination) on the uptake of other vaccines (e.g., meningococcal [2]) have limited literature. Spillover effects of one vaccine policy on the same vaccine uptake by non-target age groups also existed. For instance, the federal HPV vaccine recommendation in 2011 for men aged 11–21 had modest spillover effects, a 5.6 % increased likelihood for men aged 22–25, and 3.0 % and 1.2 % increases for older men aged 26–40 and 41–60 [3].

Yet, the spillover effects of federal pandemic vaccine recommendations, as policies, have not been examined. This is important because pandemic influenza viruses caused millions of deaths, such as SARS-CoV-2 (2019) and H1N1 (2009 and 1918). Seasonal influenza causes about 41 million illnesses, 710,000 hospitalizations, 52,000 deaths [4], and $10.4 billion in annual medical costs [5]. Seasonal and pandemic influenza vaccines trigger cross-reactive antibody responses due to binding agents [6], generating a positive spillover effect on human immunity [7,8].

Individual socioeconomic and demographic characteristics also affect vaccination. For instance, insurance reduces the real price of vaccination faced by consumers and thus may increase their likelihood of demanding vaccines. However, the degree of such increases due to policy may differ across various insurance types, age groups, racial and ethnic groups, health statuses, and child statuses, among others. Yet, the literature lacks studies exploring heterogeneity across certain characteristics (e.g., age and insurance type combined, American Indian and Alaska Native (AIAN)).

This paper examines three aims: the effectiveness of federal influenza vaccine recommendations in influenza vaccination, the spillover effect of the federal H1N1 pandemic vaccine recommendation on influenza vaccination, and any associated heterogeneous policy effects across individual characteristics. Specifically, the Centers for Disease Control and Prevention (CDC) expanded the universal seasonal influenza vaccine recommendation from younger children (6 months-4 years) to older children (5–18 years) in 2008 [9]. It introduced a universal H1N1 vaccine recommendation to children and younger adults (6 months-24 years) in 2009 [10]. Following the H1N1 pandemic, the CDC expanded the seasonal influenza vaccine recommendation to all individuals over 6 months in 2010 [11]. Findings can guide future vaccine policymaking if other types of policies are needed to target certain subpopulations to reach minimum coverage goals, and take advantage of positive spillover effects, if any, to maximize coverage.

## 2 Methods

### 2.1 Data and study population

We used 2004–2015 National Health Interview Survey (NHIS) data, pooled cross-sectional surveys administered by the CDC through stratified multistage sampling, collecting respondent vaccination status, insurance types, demographic, and socioeconomic characteristics [12]. The study period included five years before and after each of the three policies. Because each policy was introduced early in the year but implemented during the influenza season near the end of the year, while annual NHIS interviews took place in August, we considered the year a policy was introduced to be the last pre-policy year. For example, for the policy introduced in early 2008 and implemented later in the 2008 influenza season (October 2008–April 2009 [9]), the pre-policy period included 2004–2008 because NHIS interviews occurred in August 2008. Accordingly, the post-policy period spanned 2009–2013. Each before-after comparison assessed changes in vaccination following each policy implementation. We used population-level vaccination rate and coverage interchangeably and distinguished them from individual-level vaccination status or probability.

### 2.2 Statistical analysis and variables

To estimate the effects of each policy, we utilized a Linear Probability Model (LPM) with fixed effects, specified as follows:

$$V_{it}=\alpha +\beta \times \left[\text{Year}\;T\;\text{Policy}\;\text{Dummy}\right]+X_{it}\theta +\delta_{r}+\eta_{a}+\varepsilon_{ait.}$$

The outcome variable, *V*_*it*_, is the influenza vaccination status for individual *i* in year *t*. This dummy variable (dummy hereafter) takes value 1 if an individual received an influenza vaccine (shots, sprays, or both) within the past 12 months, and value 0 otherwise. The outcome variable for each policy focuses on vaccination status by each targeted age group: The 2008, 2009, and 2010 policies targeted 5–18, 19–24, and 25 years and above age groups, respectively.

The main independent variable is Year *T* Policy Dummy, where *T* = {2008, 2009, 2010}. It takes value 1 from the year *T* + 1 to the end of the study period and 0 otherwise. For example, for the policy introduced in early 2008, *T* is 2008; it takes value 1 for years 2009–2013 and value 0 for years 2004–2008. The coefficient *β* measures the policy effect on the influenza vaccination probability of an individual.

*X*_*it*_ denotes a set of control variables (controls hereafter), including insurance type, number of children, self-reported health status, gender, education, family income, and race and ethnicity, including White African American, Asian American, American Indian and Alaska Native (AIAN), and Hispanic American. *η*_*a*_ denotes age-fixed effects, addressing any time-invariant age-specific unobserved heterogeneity. *δ*_*r*_ represents region-fixed effects, addressing time-invariant region-level unobserved factors that affect individual influenza vaccination, such as long-term weather conditions. Four census regions include the Northeast (default), Midwest, South, and West.

Furthermore, we examined heterogeneous policy effects to measure how much the effect of each policy varied across each individual characteristic, using interaction terms between each policy dummy and each control. Meanwhile, estimates of single policy dummies served as a sensitivity analysis.

The inclusion of controls and fixed effects mitigated concerns about omitted variable bias and other potential confounding factors. It is impossible to control for all individual-level unobserved characteristics unavailable in data, such as risk preference, social and family influence, and time-varying characteristics. Also, individual-level unobserved characteristics are usually not systematically correlated with the timing of when the federal government introduces a policy. This mitigated endogeneity concerns. Results should be interpreted with caution, as the observational study estimates association rather than causality.

## 3 Results

### 3.1 Descriptive results

Table 1 summarizes data on 77,361, 23,653, and 238,866 individuals in age groups targeted by the 2008, 2009, and 2010 policies, respectively. Given each policy in each panel, it compares pre- (first column) and post-policy (second column) mean differences (third column) with t statistics in parentheses, whose value greater than 2 was considered statistically significant. In Panel A, the 2008 policy-targeted child group showed that its population influenza vaccination rate drastically increased from pre-policy 0.10 to post-policy 0.38. Similarly, in Panel B, the 2009 policy-targeted younger adult group revealed a substantial increase in influenza vaccination coverage from pre-policy 0.13 to post-policy 0.24. Panel C shows a relatively small rise from pre-policy 0.35 to post-policy 0.43 among the 2010 policy-targeted older adult group. All three differences were statistically significant, signaling potential policy effects and motivating subsequent empirical estimations. Below, we report statistically significant results with P < 0.01 unless otherwise specified.

**Table 1:** Summary statistics (mean and standard deviation) of influenza vaccination rates and demographic and socioeconomic characteristics by federal vaccine recommendation age group.

	2008 Policy: 5–18 Years Old			2009 Policy: 19–24 Years Old			2010 Policy: 25 Years Old and Older		
	2004–2008	2009–2013	Diff (tstat)	2005–2009	2010–2014	Diff (tstat)	2005–2011	2011–2015	Diff (tstat)
Influenza Vaccination Rate	0.100	0.380	0.274	0.130	0.240	0.104	0.350	0.430	0.084
	(0.300)	(0.480)	(95.090)	(0.340)	(0.420)	(20.515)	(0.480)	(0.500)	(41.637)
Gender									
Male	0.510	0.520	0.002	0.460	0.460	0.007	0.440	0.450	0.005
	(0.500)	(0.500)	(0.634)	(0.500)	(0.500)	(1.027)	(0.500)	(0.500)	(2.660)
Children									
Children Under 5 Years Old	0.003	0.005	−0.002	0.200	0.180	−0.023	0.120	0.110	−0.010
	(0.007)	(0.006)	(-3.593)	(0.400)	(0.380)	(-4.529)	(0.320)	(0.310)	(-7.403)
Self-Reported Health Status									
Excellent/Very Good	0.790	0.810	0.012	0.750	0.750	−0.002	0.560	0.560	−0.004
	(0.400)	(0.400)	(3.996)	(0.430)	(0.430)	(-0.304)	(0.500)	(0.500)	(-2.160)
Fair/Poor	0.020	0.020	0.002	0.040	0.050	0.002	0.160	0.160	0.003
	(0.150)	(0.160)	(1.532)	(0.210)	(0.210)	(0.909)	(0.370)	(0.370)	(1.852)
Education									
High School				0.270	0.270	−0.004	0.270	0.250	−0.018
				(0.440)	(0.440)	(-0.614)	(0.440)	(0.430)	(-9.626)
Some College				0.450	0.480	0.028	0.280	0.290	0.015
				(0.500)	(0.500)	(4.277)	(0.450)	(0.450)	(7.977)
College and Above				0.120	0.140	0.020	0.270	0.300	0.025
				(0.330)	(0.350)	(4.398)	(0.450)	(0.460)	(13.136)
Family Income									
100K and Above	0.160	0.220	0.063	0.080	0.070	−0.003	0.160	0.180	0.022
	(0.360)	(0.410)	(22.672)	(0.270)	(0.260)	(0.758)	(0.360)	(0.380)	(14.219)
Race									
African American	0.180	0.180	0.004	0.180	0.170	−0.011	0.160	0.150	−0.014
	(0.380)	(0.380)	(1.394)	(0.380)	(0.370)	(−2.199)	(0.370)	(0.360)	(-9.475)
American Indian/Alaska Native	0.010	0.010	0.002	0.010	0.010	0.001	0.010	0.010	0.002
	(0.110)	(0.110)	(2.340)	(0.110)	(0.110)	(0.472)	(0.090)	(0.100)	(4.222)
Asian	0.040	0.060	0.022	0.060	0.070	0.014	0.060	0.060	0.003
	(0.200)	(0.240)	(13.853)	(0.240)	(0.260)	(4.383)	(0.230)	(0.240)	(3.120)
Hispanic	0.290	0.300	0.016	0.250	0.230	−0.019	0.170	0.170	−0.008
	(0.450)	(0.460)	(4.934)	(0.430)	(0.420)	(-3.337)	(0.380)	(0.370)	(-5.406)
Insurance									
Public Insurance	0.270	0.350	0.086	0.130	0.160	0.028	0.110	0.140	0.031
	(0.440)	(0.480)	(25.922)	(0.340)	(0.370)	(6.060)	(0.310)	(0.350)	(22.323)
Private Insurance	0.590	0.530	−0.063	0.520	0.560	0.041	0.620	0.590	−0.028
	(0.490)	(0.500)	(-17.781)	(0.500)	(0.500)	(6.313)	(0.490)	(0.490)	(-13.776)
Children Insurance	0.060	0.070	0.014	0.000	0.000	0.000	0.000	0.000	−0.001
	(0.230)	(0.260)	(8.175)	(0.005)	(0.005)	(-0.035)	(0.004)	(0.003)	(-3.695)
Military Insurance	0.020	0.020	0.001	0.030	0.030	0.003	0.040	0.040	0.006
	(0.140)	(0.140)	(1.236)	(0.160)	(0.170)	(1.480)	(0.190)	(0.210)	(7.414)
Number of Observations	40,583	36,778		10,590	13,063		99,622	139,244	

### 3.2 2008 influenza vaccine policy effectiveness: 5-18-year age group

Table 2, Table 3, Table 4 show statistically significant positive impacts of all three policies on influenza vaccination probability in their targeted age groups. Table 2, row 1, reports LPM estimates of the 2008 policy effectiveness. Column 1 estimates the *β* coefficient as 0.274, indicating that 5- to 18-year-old children were 27.4 % more likely to be vaccinated due to the policy. The coefficient estimates remain stable, ranging from 0.265 to 0.275 after adding fixed effects and controls.

**Table 2:** Effectiveness of the federal 2008 influenza vaccine recommendation on individual influenza vaccination likelihood, by individual-level characteristics, with age and region fixed effects.

Dependent Variable: Vaccination Status for Individuals Aged 5-18										
	(1)	(2)	(3)	(4)	(5)	(6)	(7)	(8)	(9)	(10)
2008 Policy	0.274∗∗∗	0.273∗∗∗	0.275∗∗∗	0.274∗∗∗	0.274∗∗∗	0.274∗∗∗	0.272∗∗∗	0.269∗∗∗	0.267∗∗∗	0.265∗∗∗
	(0.002)	(0.014)	(0.014)	(0.014)	(0.014)	(0.014)	(0.014)	(0.014)	(0.013)	(0.014)
Male					−0.002	−0.002	−0.002	−0.002	−0.002	−0.002
					(0.003)	(0.003)	(0.003)	(0.003)	(0.003)	(0.003)
Health Status										
Excellent/VeryGood						−0.016∗∗∗	−0.021∗∗∗	−0.017∗∗∗	−0.019∗∗∗	−0.015∗∗∗
						(0.004)	(0.003)	(0.003)	(0.003)	(0.003)
Fair						0.070∗∗∗	0.072∗∗∗	0.072∗∗∗	0.070∗∗∗	0.067∗∗∗
						(0.011)	(0.011)	(0.011)	(0.011)	(0.011)
Income										
Income More than 100K							0.046∗∗∗	0.049∗∗∗	0.045∗∗∗	0.051∗∗∗
Race							(0.007)	(0.006)	(0.006)	(0.006)
American Indian										
/Alaska Native								0.068∗∗∗ (0.011)	0.075∗∗∗ (0.011)	0.070∗∗∗ (0.012)
African American								0.019∗∗∗	0.018∗∗∗	0.013∗∗∗
								(0.004)	(0.004)	(0.003)
Asian								0.083∗∗∗	0.085∗∗∗	0.085∗∗∗
								(0.007)	(0.007)	(0.008)
Hispanic								0.021∗∗∗	0.031∗∗∗	0.025∗∗∗
								(0.004)	(0.004)	(0.003)
Insurance										
Public Insurance										0.081∗∗∗
										(0.003)
Private Insurance										0.053∗∗∗
										(0.006)
Children Insurance										−0.013∗∗
										(0.004)
Military Insurance										0.104∗∗∗
										(0.007)
No Insurance									−0.081∗∗∗	
									(0.004)	
Age FE	No	Yes	No	Yes	Yes	Yes	Yes	Yes	Yes	Yes
Region FE	No	No	Yes	Yes	Yes	Yes	Yes	Yes	Yes	Yes
R-squared	0.105	0.114	0.108	0.117	0.117	0.118	0.120	0.122	0.126	0.126
Observations	77,361	77,361	77,361	77,361	77,361	77,361	77,361	77,361	77,361	77,361

**Table 3:** Spillover effects of the federal 2009H1N1 vaccine recommendation on individual influenza vaccination likelihood, by individual-level characteristics, with age and region fixed effects.

Dependent Variable: Vaccination Status for Individuals Aged 19-24											
	(1)	(2)	(3)	(4)	(5)	(6)	(7)	(8)	(9)	(10)	(11)
2009 Policy	0.104∗∗∗	0.104∗∗∗	0.105∗∗∗	0.105∗∗∗	0.106∗∗∗	0.106∗∗∗	0.103∗∗∗	0.103∗∗∗	0.103∗∗∗	0.098∗∗∗	0.098∗∗∗
	(0.004)	(0.004)	(0.004)	(0.004)	(0.004)	(0.004)	(0.004)	(0.004)	(0.004)	(0.004)	(0.005)
Male					−0.052∗∗∗	−0.052∗∗∗	−0.043∗∗∗	−0.043∗∗∗	−0.044∗∗∗	−0.038∗∗∗	−0.038∗∗∗
					(0.006)	(0.006)	(0.006)	(0.006)	(0.006)	(0.005)	(0.005)
Children Under 5 Years Old					0.011	0.012	0.038∗∗∗	0.039∗∗∗	0.041∗∗∗	0.035∗∗∗	0.035∗∗∗
					(0.007)	(0.007)	(0.007)	(0.007)	(0.007)	(0.007)	(0.008)
Health Status											
Excellent/VeryGood						0.015∗∗	0.004	0.003	0.004	−0.001	−0.001
						(0.005)	(0.004)	(0.004)	(0.004)	(0.004)	(0.004)
Fair						0.020∗∗	0.027∗∗	0.027∗∗	0.028∗∗	0.024∗∗	0.024∗∗
						(0.007)	(0.007)	(0.007)	(0.007)	(0.007)	(0.007)
Education											
High School							0.006	0.005	0.005	−0.004	−0.004
							(0.007)	(0.007)	(0.007)	(0.008)	(0.008)
Some College							0.049∗∗∗	0.048∗∗∗	0.046∗∗∗	0.022∗∗∗	0.021∗∗∗
							(0.006)	(0.006)	(0.005)	(0.004)	(0.005)
College and Above							0.094∗∗∗	0.093∗∗∗	0.087∗∗∗	0.052∗∗∗	0.054∗∗∗
							(0.012)	(0.012)	(0.010)	(0.012)	(0.012)
Income											
Income More than 100K								0.031∗∗	0.030∗∗	0.020∗	0.021∗
								(0.010)	(0.010)	(0.009)	(0.009)
Race											
American Indian									0.040	0.058∗∗	0.056∗
/Alaska Native									(0.021)	(0.022)	(0.022)
African American									−0.005	−0.001	0.000
									(0.009)	(0.009)	(0.010)
Asian									0.040∗∗∗	0.042∗∗∗	0.041∗∗∗
									(0.006)	(0.005)	(0.004)
Hispanic									−0.002	0.015∗	0.015∗
									(0.006)	(0.006)	(0.006)
Insurance											
No Insurance										−0.095∗∗∗	
										(0.003)	
Public Insurance											0.079∗∗∗
											(0.008)
Private Insurance											0.082∗∗∗
											(0.002)
Children Insurance											0.023
											(0.066)
Military Insurance											0.189∗∗∗
											(0.025)
Age FE	No	Yes	No	Yes	Yes	Yes	Yes	Yes	Yes	Yes	Yes
Region FE	No	No	Yes	Yes	Yes	Yes	Yes	Yes	Yes	Yes	Yes
R-squared	0.017	0.018	0.020	0.020	0.025	0.025	0.030	0.031	0.031	0.040	0.041
Observations	23,653	23,653	23,653	23,653	23,653	23,653	23,653	23,653	23,653	23,653	23,653

**Table 4:** Effectiveness of the federal 2010 influenza vaccine recommendation on individual influenza vaccination likelihood, by individual-level characteristics, with age and region fixed effects.

Dependent Variable: Vaccination Status for Individuals Aged 25 and Above											
	(1)	(2)	(3)	(4)	(5)	(6)	(7)	(8)	(9)	(10)	(11)
2010 Policy	0.084∗∗∗	0.070∗∗∗	0.085∗∗∗	0.071∗∗∗	0.072∗∗∗	0.072∗∗∗	0.067∗∗∗	0.065∗∗∗	0.065∗∗∗	0.065∗∗∗	0.066∗∗∗
	(0.002)	(0.003)	(0.003)	(0.003)	(0.003)	(0.003)	(0.003)	(0.003)	(0.003)	(0.003)	(0.003)
Male					−0.055∗∗∗	−0.055∗∗∗	−0.054∗∗∗	−0.057∗∗∗	−0.058∗∗∗	−0.053∗∗∗	−0.059∗∗∗
					(0.004)	(0.004)	(0.004)	(0.004)	(0.004)	(0.003)	(0.003)
Children Under 5 Years Old					0.065∗∗∗	0.066∗∗∗	0.071∗∗∗	0.066∗∗∗	0.068∗∗∗	0.063∗∗∗	0.065∗∗∗
					(0.005)	(0.006)	(0.003)	(0.003)	(0.003)	(0.003)	(0.003)
Health Status											
Excellent/Very Good						0.001	−0.022∗∗∗	−0.028∗∗∗	−0.031∗∗∗	−0.034∗∗∗	−0.034∗∗∗
						(0.003)	(0.002)	(0.002)	(0.003)	(0.003)	(0.003)
Fair						0.027∗∗∗	0.044∗∗∗	0.048∗∗∗	0.049∗∗∗	0.043∗∗∗	0.055∗∗∗
						(0.003)	(0.003)	(0.003)	(0.003)	(0.003)	(0.004)
Education											
High School							0.034∗∗∗	0.031∗∗∗	0.020∗∗∗	0.009∗∗	0.004
							(0.003)	(0.003)	(0.002)	(0.003)	(0.003)
Some College							0.082∗∗∗	0.075∗∗∗	0.063∗∗∗	0.044∗∗∗	0.038∗∗∗
							(0.002)	(0.002)	(0.002)	(0.003)	(0.003)
College and Above							0.162∗∗∗	0.139∗∗∗	0.121∗∗∗	0.093∗∗∗	0.088∗∗∗
							(0.002)	(0.002)	(0.003)	(0.003)	(0.003)
Income											
Income More than 100K								0.084∗∗∗	0.078∗∗∗	0.061∗∗∗	0.062∗∗∗
								(0.003)	(0.003)	(0.003)	(0.003)
Race											
American Indian									0.036∗∗∗	0.050∗∗∗	0.048∗∗∗
/Alaska Native									(0.009)	(0.009)	(0.009)
African American									−0.060∗∗∗	−0.059∗∗∗	−0.054∗∗∗
									(0.006)	(0.006)	(0.006)
Asian									0.009∗∗	0.011∗∗	0.013∗∗∗
									(0.004)	(0.004)	(0.004)
Hispanic									−0.042∗∗∗	−0.020∗∗∗	−0.025∗∗∗
									(0.005)	(0.005)	(0.005)
Insurance											
No Insurance										−0.166∗∗∗	
										(0.007)	
Public Insurance											0.076∗∗∗
											(0.006)
Private Insurance											0.115∗∗∗
											(0.004)
Children Insurance											0.012
											(0.030)
Military Insurance											0.180∗∗∗
											(0.008)
Age FE	No	Yes	No	Yes	Yes	Yes	Yes	Yes	Yes	Yes	Yes
Region FE	No	No	Yes	Yes	Yes	Yes	Yes	Yes	Yes	Yes	Yes
R-squared	0.007	0.115	0.009	0.116	0.120	0.121	0.134	0.138	0.140	0.148	0.147
Observations	238,866	238,866	238,866	238,866	238,866	238,866	238,866	238,866	238,866	238,866	238,866

Children with military, public, and private insurance were 10.4 %, 8.1 %, and 5.3 % more likely to be vaccinated than the uninsured. Children in all other racial and ethnic groups were more likely than White children to receive influenza vaccination, with 8.5 %, 7.0 %, 2.5 %, and 1.3 % higher likelihoods for Asian, AIAN, Hispanic, and African Americans, respectively. Compared to children with poor health, those with fair health were 6.7 % more likely to be vaccinated, while those with excellent or very good health were 1.5 % less likely. Children from higher-income families were 4.5–5.1 % more likely to be vaccinated compared to those from families with income below $100,000.

### 3.3 2009 H1N1 vaccine policy spillover effect: 19-24-year age group

Table 3 reports a 9.8–10.6 % increase in influenza vaccination likelihood among younger adults due to the 2009H1N1 vaccine policy, a spillover effect smaller than the direct effect of the 2008 influenza vaccine policy, with a 26–28 % such increase among children. Younger adults with military, public, and private insurance were 18.9 %, 7.9 %, and 8.2 %, respectively, more likely than the uninsured to vaccinate, with the publicly insured slightly lower than the privately insured. Those with young children were 3.5 % more likely to vaccinate than those without. Those with fair health were 5.5 % more likely to vaccinate than those with poor health. Males were 3.8 % less likely than females to vaccinate. Younger adults with a college education, some college education, and an income above $100,000 were 5.4 %, 2.1 %, and 2.1 % more likely to vaccinate, respectively, than those with lower education and income levels.

### 3.4 2010 influenza vaccine policy effectiveness: 25 years and above age group

Table 4 presents a 6.5–8.5 % increase in vaccination likelihood among older adults due to the 2010 policy, which is smaller than the 26–28 % and 10 % increases observed with the 2008 and 2009 policies. This signals that older adults were relatively less responsive to the federal vaccine recommendation. Older adults with military, public, and private insurance were 18 %, 7.6 %, and 11.5 %, respectively, more likely than the uninsured to vaccinate, with the publicly insured substantially lower than the privately insured. Compared to Whites, AIAN and Asian Americans were 1.3–4.8 % more likely to vaccinate, while Hispanic and African Americans were 2.5 % and 5.4 % less likely. Those with young children were 6.5 % more likely to vaccinate than those without. Interestingly and consistently, compared to those with poor health, those with fair health were 5.5 % more likely to vaccinate, but those with excellent or very good health were 3.4 % less likely. Older adults with a college education, some college education, and an income above $100,000 were 8.8 %, 3.8 %, and 6.2 % more likely to vaccinate, respectively, than those with lower education and income levels.

### 3.5 Heterogeneous policy effects

Tables S1–S3 (Supplementary Materials) examine the heterogeneous impacts of the three policies across insurance, race and ethnicity, children, gender, education, and income; that is, changes in influenza vaccination likelihood of individuals with each characteristic due to each policy. In Table S1, children with all insurance types positively responded to the 2008 influenza vaccine policy than the uninsured, with a 5.0–8.8 % higher likelihood. Asian and Hispanic American children positively responded to the policy, with 2.8 % (P < 0.05) and 2.4 % higher likelihoods. Higher-income children responded positively, but male children responded negatively to the policy, with 4.3 % higher and 1.4 % lower likelihoods, respectively.

In Table S2, younger adults across all insurance types exhibited spillover effects from the 2009H1N1 vaccine policy, with a 5.2–10.4 % higher likelihood. Asian and African American younger adults responded oppositely to the policy, with 8.2 % higher and 1.9 % lower likelihoods (P < 0.05), respectively. Higher-educated, higher-income younger adults and those with young children responded positively to the policy with 3.2–5.7 % higher likelihoods, while male younger adults responded negatively with a 4.3 % lower likelihood.

In Table S3, only privately insured older adults responded to the 2010 influenza vaccine policy, with a 2.0 % higher likelihood. No racial or ethnic group responded to the policy differently from the Whites. Again, higher-educated, higher-income older adults and those with young children responded positively to the policy with 2.6–5.4 % higher likelihoods, while males responded negatively with a 1.7 % lower likelihood. Estimates of all single policy dummies in Tables S1–S3 with heterogeneous effects shows similar statistical significance and magnitudes to those in Table 2, Table 3, Table 4, a consistent sensitivity analysis result.

## 4 Discussion

Both 2008 and 2010 influenza vaccine recommendations boosted influenza vaccination likelihood by 20.9–26.5 % among children (Table 2 and S1) and by 5.2–6.6 % among older adults (Table 4 and S2). Childhood vaccination benefits from established institutions (e.g., schools), while adults encounter decision-making barriers (e.g., personal beliefs) [13]. Older adults are further less likely to seek vaccination [14], due to low perceived need and efficacy, and high perceived safety issues [15]. They also invest less in health than younger individuals due to lower economic returns, according to the Health Capital Theory [16], a key group for intervention.

The 2009H1N1 vaccine recommendation had a positive spillover effect, with a 5.7–9.8 % increase in influenza vaccination likelihood among younger adults (Table 3 and S2). Spillover effects of one vaccine policy (e.g., Tdap) on the uptake of other vaccines existed (e.g., meningococcal, HPV [2]) through possible mechanisms (e.g., heightened awareness, provider recommendations, health system readiness). Spillover effects of one vaccine policy on the same vaccine uptake by non-target age groups also existed [3]. Spillover effects of consumer attitudes, rather than policies, toward one vaccine (e.g., COVID-19) on their attitudes toward other vaccines (e.g., influenza, MMR, HPV, chickenpox) were spotlighted during the COVID-19 pandemic [[17], [18], [19]]. Spillover effects between respiratory syncytial virus (RSV) and influenza vaccines were absent in the literature, despite high RSV vaccine hesitancy among older adults [20]. Future research may investigate mechanisms underlying such effects using new data. Future policies may consider vaccines and groups that have spillover effects to maximize coverage.

Insurance type plays a subtle yet significant role in boosting influenza vaccination. Military insurance was consistently associated with the highest likelihood of vaccination increases across all three age groups. Public insurance was associated with a higher vaccination likelihood than private and no insurance for children. Conversely, private insurance was associated with a higher likelihood than public insurance for both younger and older adults (Table 2, Table 3, Table 4). In response to each policy, military and public insurance were associated with higher vaccination likelihoods than private and no insurance for children. However, only privately insured older adults responded to the 2010 policy (Tables S1–S3). Although cost barriers were partially addressed by the Affordable Care Act (ACA)-mandated no cost-sharing for CDC-recommended vaccines, non-cost barriers were found to systematically persist at the individual, interpersonal, organizational (e.g., access to providers), community (e.g., cultural norms, information infrastructure, trust), and societal levels (e.g., structural racism) [1]. Future policies may address cost barriers of the uninsured and multi-level non-cost barriers of privately insured children and publicly insured adults.

Racial and ethnic groups exhibit different influenza vaccination likelihoods (Table 2, Table 3, Table 4) and respond distinctly to different vaccine policies (Tables S1–S3). White children have the lowest likelihood, in general (Table 2), White, African American, and AIAN children responded to the 2008 policy less than Asian and Hispanic children (Table S1). AIAN children can access free CDC-recommended vaccines through the Federal Vaccines for Children Program [21]. Moreover, compared to White older adults, Asians and AIANs have higher vaccination likelihoods, while African and Hispanic Americans have lower likelihoods, in general (Table 4). This may reflect the Asian cultural emphasis on preventive care [22] and the AIAN no-cost access through the Indian Health Service system [23]. However, African and Hispanic Americans may face societal-level barriers, such as distrust in government or health systems [24] and trust in interpersonal sources, and thus are more suitable for community-engaged interventions [25]. Furthermore, older adults of all groups did not respond to the 2010 policy differently (Table S3), and thus needed policies other than the federal recommendation. Finally, Asian and African American young adults had significant positive and negative spillover effects of the 2009H1N1 vaccine policy, respectively (Table S2). Future policymaking may consider such spillover effects of one vaccine policy to help achieve targeted coverage of another vaccine across different racial and age groups.

Adults with young children are more likely than the childless to receive influenza vaccination due to the policies, as young children are at high risk of influenza-related complications [26] and face associated healthcare costs and opportunity costs (e.g., work absenteeism, wage loss) [26,27].

Influenza vaccination likelihood decreases in order of self-reported health, from fair to poor, and then to excellent or very good health [28]. Interventions should target individuals with poor health, then individuals with very good health [1], if still not reaching the 50 % herd immunity threshold [29].

No significant gender difference existed in influenza vaccination among children but adults. Males often face personal and social barriers to seeking help and care [30,31], making them a target group for interventions.

Adults with college education or higher had a higher likelihood than high school graduates or dropouts (Table 3, Table 4, S1-S2) [32]. Interventions may educate the latter on vaccine-preventable diseases and vaccine information, as vaccine awareness decreased with low literacy [1].

Children and adults from higher-income families had higher likelihoods due to policies (Tables S1–S3). Income can affect vaccination through various mechanisms [1]. Lower-income individuals face cost, literacy, and other barriers to vaccination. Medicaid expansion, which increased eligibility for low-income individuals, may not necessarily improve influenza vaccination [33]. Simultaneously needed are anti-poverty policies.

Limitations exist. Results should be interpreted with caution, as the observational study estimated association rather than causality. The end of the study period 2004–2015 somewhat overlaps with the ACA implementation starting in 2014. However, this did not affect our evaluations of the 2008 and 2009 policies, and only somewhat affected the 2010 policy, whose post-policy period was 2011–2015, with 2011–2013 intact. NHIS data do not measure media coverage. However, as children and adults often have different media exposure, our age-fixed effects partially addressed the potential influence of media coverage. State policies could not be evaluated due to the unavailability of public data on an individual's state residency.

To conclude, this paper focuses on the national-level, inexpensive, demand-side vaccine policy—federal recommendations. Both influenza vaccine recommendations effectively boosted the vaccination likelihoods of target populations, with older adults less responsive to policies than children. H1N1 vaccine recommendation also had a spillover effect on influenza vaccination. Low influenza vaccination likelihoods exist across characteristics: Children who are uninsured or privately insured, White, and low-income; Adults who are uninsured or publicly insured, White, African and Hispanic American, male, childless, poor or excellent self-reported health, low-educated, and low-income. Future policies may address the cost barrier for the uninsured, and the multi-level non-cost barriers faced by privately insured children and publicly insured adults. Future policies may target the identified subpopulations beyond the federal recommendation, such as simultaneous anti-poverty policies, and utilize the spillover effects of one vaccine policy to maximize the coverage of other vaccines. Future research may investigate potential policy spillover effects among influenza, COVID-19, RSV, and other vaccines.

## Ethical approval

This health economic study is a secondary data analysis of existing publicly available de-identified data. In general, secondary data analyses in economics do not require IRB approval, especially if the data is publicly available, de-identified, or does not involve identifiable individuals or protected health information, which is the case in our study.

## Funding

We acknowledge the research support from the 10.13039/100001298Presbyterian Health Foundation grant number 20240169.

## Declaration of competing interest

The authors declare that they have no known competing financial interests or personal relationships that could have appeared to influence the work reported in this paper.
