# Supplementary material for: Power of public health advice: Effectiveness and spillover effects of federal vaccine recommendations
Source: Public Health Pract (Oxf). 2025 Oct 14;10:100667. doi: 10.1016/j.puhip.2025.100667 (PMC12597293; doi:10.1016/j.puhip.2025.100667)
Supplement: Multimedia component 1 [file mmc1.docx]

**Supplementary Materials**

Table S1: Heterogeneous effects of the federal 2008 influenza vaccine recommendation on individual influenza vaccination likelihood, by individual-level characteristics, with age and region fixed effects.



Dependent Variable: Vaccination Status for Individuals in Age Between 6 and 18

|  | (1) |  | (2) |  | (3) |  | (4) |  |
| --- | --- | --- | --- | --- | --- | --- | --- | --- |
| By Gender: Base Group Female |  |  |  |  |  |  |  |  |
|   2008 Policy Dummy × Male  By Race: Base Group White | -0.014** (0.005) |  |  |  |  |  |  |  |
|   2008 Policy Dummy × Hispanic |  |  | 0.024*** (0.006) |  |  |  |  |  |
| 2008 Policy Dummy × Asian |  |  | 0.028** (0.0103) |  |  |  |  |  |
| 2008 Policy Dummy × African American |  |  | -0.008 |  |  |  |  |  |
|  |  |  | (0.007) |  |  |  |  |  |
| 2008 Policy Dummy × American Indian/Alaska Native |  |  | -0.024 |  |  |  |  |  |
|  |  |  | (0.025) |  |  |  |  |  |
| By Income: Base Group Income *<* 100K |  |  |  |  |  |  |  |  |
|   2008 Policy Dummy × Income Above 100K  By Insurance: Base Group Income No Insurance |  |  |  |  | 0.043*** (0.008) |  |  |  |
|   2008 Policy Dummy × Public Insurance |  |  |  |  |  |  |  | 0.088*** (0.007) |
| 2008 Policy Dummy × Private Insurance |  |  |  |  |  |  |  | 0.050*** (0.010) |
| 2008 Policy Dummy × Children Insurance |  |  |  |  |  |  |  | -0.011 |
|  |  |  |  |  |  |  |  | (0.010) |
| 2008 Policy Dummy × Military Insurance |  |  |  |  |  |  |  | 0.085*** (0.017) |
| 2008 Policy Dummy | 0.273*** |  | 0.259*** |  | 0.257*** |  |  | 0.209*** |
|  | (0.014) |  | (0.015) |  | (0.013) |  |  | (0.013) |
| All Other Controls | Yes |  | Yes |  | Yes |  |  | Yes |
| Age FE | Yes |  | Yes |  | Yes |  |  | Yes |
| Region FE | Yes |  | Yes |  | Yes |  |  | Yes |
| R-squared | 0.126 |  | 0.126 |  | 0.127 |  |  | 0.127 |
| Observations | 77,361 |  | 77,362 |  | 77,363 |  |  | 77,364 |

Sources: 2004-2015 National Health Interview Survey (NHIS) nationally representative data. Notes: The 2008 Policy Dummy variable that takes the value 1 in post policy period and 0 otherwise. The numbers in the first parenthesis of each column represent the region-level level clustered robust standard errors. The notation ∗ represents the statistical significance levels:

∗ *p <* 0*.*10, ∗ ∗ *p <* 0*.*05 and ∗ ∗ ∗ *p <* 0*.*01.

Table S2: Heterogeneous spillover effects of the federal 2009 H1N1 vaccine recommendation on individual influenza vaccination likelihood, by individual-level characteristics, with age and region fixed effects.



Dependent Variable: Vaccination Status for Individuals Aged 19-24

|  | (1) | (2) | (3) | (4) | (5) | (6) |
| --- | --- | --- | --- | --- | --- | --- |
| By Gender: Base Group Female |  |  |  |  |  |  |
|   2009 Policy Dummy × Male  By Family Status: Base Group No Children | -0.043*** (0.006) |  |  |  |  |  |
|   2009 Policy Dummy × Having Children Under 5 Years Old |  | 0.032** (0.008) |  |  |  |  |
| By Race: Base Group White |  |  |  |  |  |  |
|   2009 Policy Dummy × African American |  |  | -0.019** (0.007) |  |  |  |
| 2009 Policy Dummy × Asian |  |  | 0.082** (0.025) |  |  |  |
| 2009 Policy Dummy × Hispanic |  |  | 0.004 |  |  |  |
|  |  |  | (0.006) |  |  |  |
| 2009 Policy Dummy × American Indian/ Alaska Native |  |  | 0.058 |  |  |  |
|  |  |  | (0.029) |  |  |  |
| By Education: Base Group HS Dropout |  |  |  |  |  |  |
|   2009 Policy Dummy × HS |  |  |  | 0.011 |  |  |
|  |  |  |  | (0.019) |  |  |
| 2009 Policy Dummy × Some College |  |  |  | 0.033 |  |  |
|  |  |  |  | (0.019) |  |  |
| 2009 Policy Dummy × College and Above |  |  |  | 0.057*** (0.006) |  |  |
| By Education: Base Group Income *<* 100K |  |  |  |  |  |  |
|   2009 Policy Dummy × Income Above 100K  By Insurance: Base Group Income No Insurance |  |  |  |  | 0.057* (0.024) |  |
|   2009 Policy Dummy x Public Insurance |  |  |  |  |  | 0.069*** (0.007) |
| 2009 Policy Dummy × Private Insurance |  |  |  |  |  | 0.052*** (0.009) |
| 2009 Policy Dummy × Children Insurance |  |  |  |  |  | 0.123 |
|  |  |  |  |  |  | (0.122) |
| 2009 Policy Dummy × Military Insurance |  |  |  |  |  | 0.104** (0.034) |
| 2009 Policy Dummy | 0.118*** | 0.091*** | 0.094*** | 0.071*** | 0.093*** | 0.057*** |
|  | (0.004) | (0.005) | (0.005) | (0.010) | (0.003) | (0.004) |
| All Other Controls | Yes | Yes | Yes | Yes | Yes | Yes |
| Age FE | Yes | Yes | Yes | Yes | Yes | Yes |
| Region FE | Yes | Yes | Yes | Yes | Yes | Yes |
| R-squared | 0.044 | 0.044 | 0.044 | 0.044 | 0.044 | 0.045 |
| Observations | 23,653 | 23,653 | 23,653 | 23,653 | 23,653 | 23,653 |

Sources: 2004-2015 National Health Interview Survey (NHIS) nationally representative data. Notes: The 2009 Policy Dummy variable that takes the value 1 in post policy period and 0 otherwise. The numbers in the first parenthesis of each column represent the region-level level clustered robust standard errors. The notation ∗ represents the statistical significance levels: ∗ *p <* 0*.*10, ∗ ∗ *p <* 0*.*05 and ∗ ∗ ∗ *p <* 0*.*01.

Table S3: Heterogeneous effects of the federal 2010 influenza vaccine recommendation on individual influenza vaccination likelihood, by individual-level characteristics, with age and region fixed effects.



Dependent Variable: Vaccination Status for Individuals Aged 25 and Above

| (1) | | (2) | (3) | (4) | (5) | (6) |
| --- | --- | --- | --- | --- | --- | --- |
| By Gender: Base Group Female |  |  |  |  |  |  |
|   2010 Policy Dummy × Male  By Family Status: Base Group No Children | -0.017*** (0.004) |  |  |  |  |  |
|   2010 Policy Dummy × Having Children Under 5 Years Old |  | 0.054*** (0.005) |  |  |  |  |
| By Race: Base Group White |  |  |  |  |  |  |
|   2010 Policy Dummy × American Indian/Alaska Native |  |  | 0.001 |  |  |  |
|  |  |  | (0.020) |  |  |  |
| 2010 Policy Dummy × African American |  |  | -0.001 |  |  |  |
|  |  |  | (0.005) |  |  |  |
| 2010 Policy Dummy × Asian |  |  | 0.012 |  |  |  |
|  |  |  | (0.009) |  |  |  |
| 2010 Policy Dummy × Hispanic |  |  | 0.004 |  |  |  |
|  |  |  | (0.004) |  |  |  |
| By Education: Base Group HS Dropout |  |  |  |  |  |  |
|   2010 Policy Dummy × HS |  |  |  | -0.005 |  |  |
|  |  |  |  | (0.006) |  |  |
| 2010 Policy Dummy × Some College |  |  |  | 0.003 |  |  |
|  |  |  |  | (0.005) |  |  |
| 2010 Policy Dummy × College and Above  By Education: Base Group Income *<* 100K |  |  |  | 0.026*** (0.006) |  |  |
|   2010 Policy Dummy × Income Above 100K  By Insurance: Base Group Income No Insurance |  |  |  |  | 0.040*** (0.005) |  |
|   2010 Policy Dummy × Public Insurance |  |  |  |  |  | 0.008 |
|  |  |  |  |  |  | (0.008) |
| 2010 Policy Dummy × Private Insurance |  |  |  |  |  | 0.020*** (0.005) |
| 2010 Policy Dummy × Children Insurance |  |  |  |  |  | -0.001 |
|  |  |  |  |  |  | (0.058) |
| 2010 Policy Dummy × Military Insurance |  |  |  |  |  | -0.007 |
|  |  |  |  |  |  | (0.012) |
| 2010 Policy Dummy | 0.074*** | 0.060*** | 0.065*** | 0.059*** | 0.059*** | 0.052*** |
|  | (0.004) | (0.003) | (0.003) | (0.006) | (0.003) | (0.003) |
| All Other Controls | Yes | Yes | Yes | Yes | Yes | Yes |
| Age FE | Yes | Yes | Yes | Yes | Yes | Yes |
| Region FE | Yes | Yes | Yes | Yes | Yes | Yes |
| R-squared | 0.151 | 0.151 | 0.151 | 0.151 | 0.151 | 0.151 |
| Observations | 234,186 | 234,186 | 234,186 | 234,186 | 234,186 | 234,186 |

Sources: 2004-2015 National Health Interview Survey (NHIS) nationally representative data. Notes: The 2010 Policy Dummy variable that takes the value 1 in post policy period and 0 otherwise. The numbers in the first parenthesis of each column represent the region-level level clustered robust standard errors. The notation ∗ represents the statistical significance levels: ∗ *p <* 0*.*10, ∗ ∗ *p <* 0*.*05 and ∗ ∗ ∗ *p <* 0*.*01.
